# Ruling out Appendicitis in Children: Can We Use Clinical Prediction Rules?

**DOI:** 10.1007/s11605-018-3997-1

**Published:** 2018-10-29

**Authors:** Paul van Amstel, Ramon R. Gorter, Johanna H. van der Lee, Huib A. Cense, Roel Bakx, Hugo A. Heij

**Affiliations:** 1 Paediatric Surgical Centre of Amsterdam, Emma Children’s Hospital Amsterdam University Medical Centre, P.O. Box 22660, 1100 DD Amsterdam, The Netherlands; 2 Division Woman and Child, Amsterdam University Medical Centre, Meibergdreef 9, 1105 AZ Amsterdam, The Netherlands; 3 Division of Surgery, Red Cross Hospital, Vondellaan 13, 1942 LE Beverwijk, The Netherlands

**Keywords:** Clinical prediction rules, Appendicitis, Children

## Abstract

**Purpose:**

To identify available clinical prediction rules (CPRs) and investigate their ability to rule out appendicitis in children presenting with abdominal pain at the emergency department, and accordingly select CPRs that could be useful in a future prospective cohort study.

**Methods:**

A literature search was conducted to identify available CPRs. These were subsequently tested in a historical cohort from a general teaching hospital, comprising all children (< 18 years) that visited the emergency department between 2012 and 2015 with abdominal pain. Data were extracted from the electronic patient files and scores of the identified CPRs were calculated for each patient. The negative likelihood ratios were only calculated for those CPRs that could be calculated for at least 50% of patients.

**Results:**

Twelve CPRs were tested in a cohort of 291 patients, of whom 87 (29.9%) suffered from acute appendicitis. The Ohmann score, Alvarado score, modified Alvarado score, Pediatric Appendicitis score, Low-Risk Appendicitis Rule Refinement, Christian score, and Low Risk Appendicitis Rule had a negative likelihood ratio < 0.1. The Modified Alvarado Scoring System and Lintula score had a negative likelihood ratio > 0.1. Three CPRs were excluded because the score could not be calculated for at least 50% of patients.

**Conclusion:**

This study identified seven CPRs that could be used in a prospective cohort study to compare their ability to rule out appendicitis in children and investigate if clinical monitoring and re-evaluation instead of performing additional investigations (i.e., ultrasound) is a safe treatment strategy in case there is low suspicion of appendicitis.

## Introduction

The diagnosis of acute appendicitis in children remains challenging as symptoms can vary from mild abdominal pain to generalized peritonitis and septicemia. Historically, the diagnosis of appendicitis is mainly based upon clinical examination in combination with biochemical variables indicative for inflammation. A disadvantage of this diagnostic strategy was the relatively high negative appendectomy rate of 12.3–19%.^1, 2^ To reduce this, an evidence-based guideline was proposed in 2010 by the Association of Surgeons of the Netherlands, which makes preoperative imaging mandatory in patients with suspected appendicitis.^3^ Ultrasound is the preferred initial diagnostic imaging modality in both the adult and pediatric population.^3^ Implementation of this guideline resulted in a significant decrease of negative appendectomies to 2.2%–5%.^2,4^ Currently in the Netherlands, in 99.7% of the adult patients’ preoperative imaging studies are performed.^4^

A consequence of the abovementioned policy is that the threshold to perform additional imaging studies is low in children presenting at the ER, especially since ultrasonography (US) can be performed quickly with minimal burden and harm for the patient. The downside of this lower threshold is the risk of potential inconclusive results from ultrasound, which may lead to exposure of children to harmful and expensive diagnostic procedures, such as CT scans, MRIs, or even diagnostic laparoscopies.^5–7^ Instead of these invasive diagnostic procedures, literature suggests that watchful waiting could be considered after non-visualization of the appendix on ultrasound.^8^ Selection of patients with high probability of acute appendicitis would help to reduce exposure to abovementioned invasive diagnostic procedures. Clinical prediction rules (CPR), such as the Alvarado score,^9^ were initially designed to diagnose appendicitis, but may also be used to rule out appendicitis. CPRs mostly consist of variables from medical history, physical examination, and biochemical testing. Large heterogeneity exists between CPRs in terms of included variables and cutoff values. Several studies showed that the value of these CPRs to diagnose appendicitis is low, reflected by positive likelihood ratios ranging from 1.7–8.5.^10–15^ Data regarding their value in ruling out appendicitis in the pediatric population are scarce.^10,16^

The first objective of this study was to identify commonly applied CPRs through a literature search. The aim of the second part of the study was to investigate the value of the identified CPRs in ruling out appendicitis in the pediatric population in the Netherlands based on the negative likelihood ratios and thereby select CPRs that could potentially be used in a future prospective cohort study. Additionally, in order to determine if the use of imaging modalities could be reduced by adopting CPRs to rule out appendicitis, we determined the number of imaging procedures performed in patients that were qualified as low risk for the disease according to these CPRs.

## Methods

### Identification of the CPRs: Literature Review

Initially, a literature search (according to the PRISMA guidelines) was performed in the PubMed database to identify potential usable CPRs.^17^ (Appendix 1) Studies were screened for title and abstract and subsequently assessed for full text by two independent reviewers. Disagreements were solved by consensus. In addition, references from the included articles were screened to identify other CPRs. No other databases than PubMed were screened for potential CPRs. Studies about CPRs that were developed to diagnose or exclude appendicitis were included in this review. A CPR was excluded if it contained variables only applicable to the adult population (e.g., points attributed to age > 50 years). CPRs consisting of more than 15 variables or variables that needed multiplication were considered as impractical in an emergency department and therefore were excluded. CPRs described in other languages than English and CPRs containing variables that were not routinely determined in our hospital, such as rectal-axillary temperature difference, were also excluded.

### Study Design and Selection of Participants

A single-center historical cohort study was conducted in a general teaching hospital. All children younger than 18 years presenting at the emergency department between January 1st 2012 and December 31st 2014 with abdominal pain were eligible for inclusion. A consecutive sample of patients with a differential diagnosis of appendicitis, identified using the international classification of diseases (ICD) codes for acute abdomen, acute appendicitis, and general abdominal complaints was used. The treating physician assigned these codes at the time of presentation at the emergency department. Children with abdominal pain due to trauma, presentation of another main complaint than abdominal pain, those not co-operating with physical examination, and those referred to another hospital were excluded.

#### Data Extraction

Data were extracted from electronic patient files using a standardized form (Appendix 2), based upon the variables used in the identified CPRs. One author (PA) performed the data extraction and 10% of the database was randomly reviewed for completeness by another author (RG). Information on the following variables was extracted:

##### General

Gender, age (years), and date of presentation.

##### Clinical Variables

Duration of abdominal pain (hours), location of pain in right iliac fossa (RIF), migration of pain, anorexia, nausea, vomiting, intensity of pain (Numeric Rating Scale (NRS)), progression of pain, steady/constant pain, hopping tenderness, coughing tenderness, percussion tenderness, dysuria, rebound tenderness, guarding, Rovsing’s sign (contralateral palpation tenderness), presence of bowel sounds, rigidity, tenderness inside/outside the right iliac fossa (RIF), and temperature.

##### Biochemical Variables

Leucocytes (×10^9^/L), C-reactive protein (CRP) (mg/L), leucocytes differential count, and urinalysis (for detection of urinary tract infection (UTI)).

##### Imaging Variables

Free fluid on ultrasonography (US), appendicolith on US, appendicular wall thickening (wall thickness > 0.7 cm) on US, appendicular abscess or suppuration on US, performance of computed tomography (CT) abdomen, and performance of magnetic resonance imaging (MRI) abdomen.

The following definitions were used in this study.

##### Appendicitis

Intraoperative diagnosis made by the treating surgeon in combination with pathologically proven inflammation of the appendix was used as the reference standard. Patients with radiographically documented appendicitis who were managed by antibiotics alone did not have pathology reports and were therefore excluded from this study.^18^

##### Non-appendicitis

No recurrence of abdominal pain or diagnosis/treatment for appendicitis by 30 days after initial presentation without any specific treatment for appendicitis. Readmission was checked for the follow-up of all cases of non-appendicitis. Telephone follow-up was not performed and we included any patient that did not subsequently return to our hospital for re-evaluation as non-appendicitis. Children with negative appendectomy were classified as non-appendicitis as well.

CPR scores were only calculated if all of the required variables were included in the patients’ records. CPRs were excluded from the analysis if the score could be calculated in less than 50% of the patients in the cohort. Cutoff values to rule out appendicitis, as presented in the original manuscript, were used to calculate the performance of the CPRs. When several cutoff values were reported, patients with a negative test result according to the lower original cutoff value of the CPRs were classified as low suspicion of appendicitis.

#### Data Analysis

IBM SPSS statistics version 22.0 was used for descriptive analysis of our data. The likelihood ratio of a negative test with its 95% CI was calculated for each CPR and displayed as value with 95% CI. A CPR with a value < 0.1 is considered as adequate to rule out appendicitis.^19^

Secondary outcomes in terms of sensitivity and negative predictive value are displayed as % with 95% CI. Performed imaging studies are displayed as numbers and percentages.

## Results

### Identification of CPRs: Literature Review

**Fig. 1:**
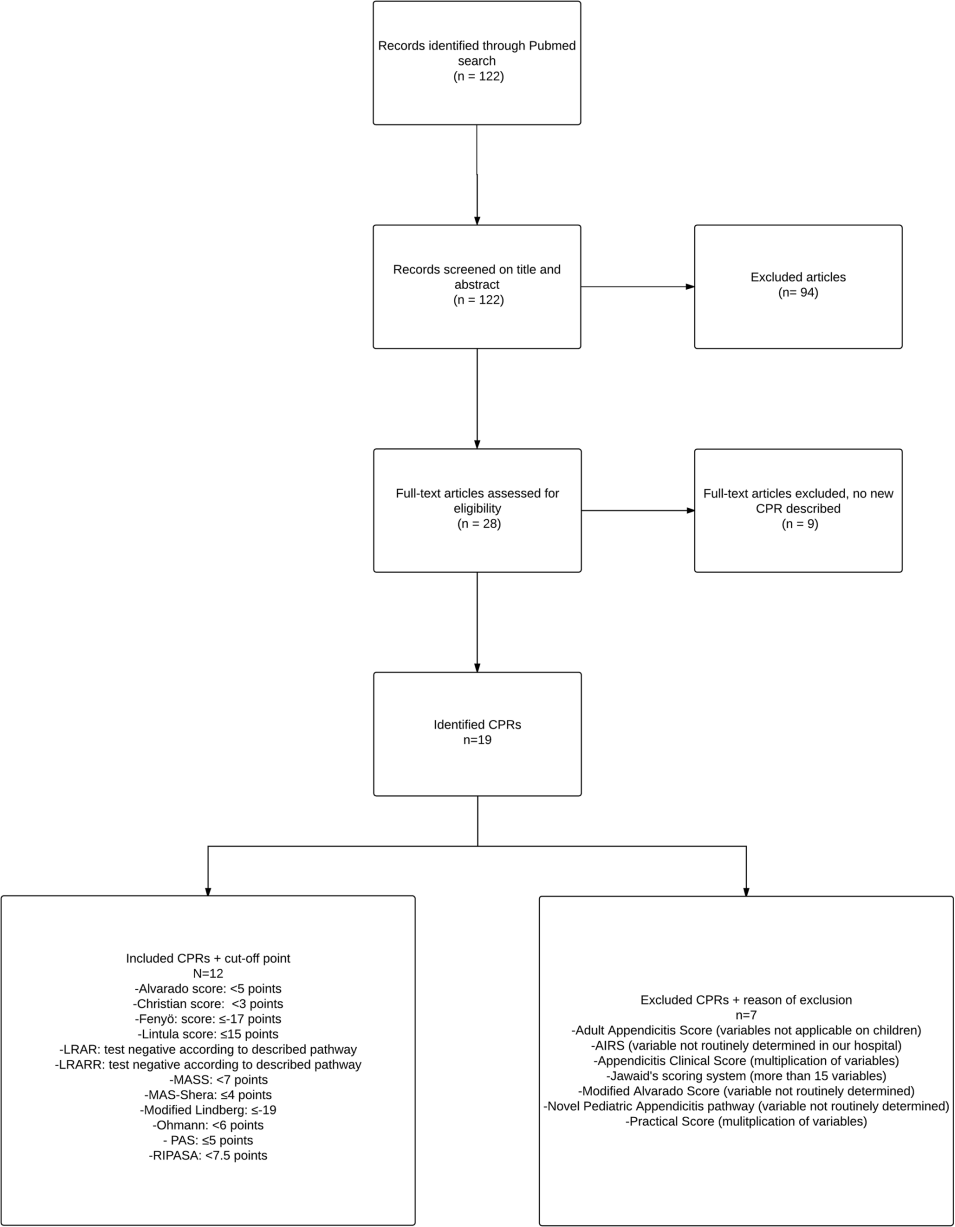
Flowchart PubMed search

In total, 19 CPRs were identified, of which seven were excluded (Fig. 1). Reasons for exclusion were multiplication of variables (*n* = 2),^20,21^ consisting of > 15 variables (*n* = 1),^22^ not applicable in children (*n* = 1),^23^ and included variables not obtained routinely (*n* = 3).^24–26^ Variables that were not routinely obtained in our hospital were a priori suspicion of appendicitis (low, intermediate, high), rectal-axillary temperature difference, and classification of rebound tenderness into light, medium, and strong.

The following 12 CPRs were included: the Alvarado score, Christian score, Fenyö score, Lintula score, Low Risk Appendicitis Rule (LRAR), Low Risk Appendicitis Rule Refinement (LRARR), Modified Alvarado Scoring System (MASS), Modified Alvarado score by Shera (MAS-Shera), modified Lindberg score, Ohmann score, Pediatric Appendicitis score (PAS), and Raja Isteri Penigran Anak Saleha Appendicitis (RIPASA) score.^9,27–37^ (Appendix 3).

### Results of the CPRs Retrospectively Tested in our Cohort

311 patients were identified in the defined time period of which 20 were excluded for the following reasons: abdominal pain caused by trauma (13 patients), presentation of another main complaint other than abdominal pain (four patients), transfer to an academic hospital (two patients), and no cooperation with physical examination (one patient).

The general characteristics of the 291 included patients are listed in Table 1. In total, 87 (29.9%) patients were diagnosed with acute appendicitis.

**Table 1:** General characteristics

Characteristic	Appendicitis	Non-appendicitis
Number of patients	87	204
Gender (male)	49 (56.3)	99 (48.5)
Age	10.8 ± 3.1*	9.9 ± 3.6*
Diagnosis		
Appendicitis	87 (100)	
Other surgical diagnosis		
Intussusception		1 (0.5)
Cholecystitis		1 (0.5)
Testicular torsion		1 (0.5)
Small bowel obstruction		1 (0.5)
Crohn’s disease		1 (0.5)
Follicular cyst		3 (1.5)
Other non-surgical diagnosis		
General abdominal pain		115 (56.4)
Gastro-enteritis		27 (13.2)
Mesenteric lymphadenitis		20 (9.8)
Fecal impaction		19 (9.3)
Urinary tract infection		6 (2.9)
Upper respiratory tract infection		4 (2.0)
Pneumonia		3 (1.5)
Enterobius vermicularis		1 (0.5)
Otitis media		1 (0.5)
Imaging		
Ultrasound	76 (87.4)	86 (42.2)
US + CT	5 (5.7)	4 (2.0)
US + MRI	4 (4.6)	10 (4.9)
No imaging	2 (2.3)	104 (51.0)

Characteristics are shown as *N* (%)

*Mean age (standard deviation)

Table 2 shows the number of patients (%) for which each CPR could be calculated. The RIPASA, modified Lindberg, and Fenyö score were excluded from further analysis as less than 50% of patients’ scores could be calculated mainly due to missing data. Aggravation with cough, progression of pain, and Rovsing’s sign were the variables with most frequently missing values for the Fenyö score, modified Lindberg, and RIPASA score, respectively. The negative likelihood ratio, sensitivity, and negative predictive value for the CPRs are presented in Table 3, which divides the CPRs into those that are developed for the pediatric population and those for the adult population. The point estimate of the negative likelihood ratio of seven CPRs was < 0.1. These were the Ohmann score (0), Alvarado score (0.03, 95% CI, 0.00–0.20), MAS-Shera (0.03, 95% C, 0.00–0.23), PAS (0.07, 95% CI, 0.00–0.22), LRARR (0.07, 95% CI, 0.02–0.23), Christian score (0.08, 95% CI, 0.00–0.22), and LRAR (0.09, 95% CI, 0.04–0.25).

**Table 2:** Number of patients from which the CPR score could be calculated

CPR	*n*/*N* (%)
LRAR	262/291 (90.0)
LRARR	260/291 (89.3)
Christian	242/291 (83.2)
Lintula	219/291 (75.3)
MASS	206/291 (70.8)
Alvarado	203/291 (69.8)
PAS	199/291 (68.4)
MAS-Shera	199/291 (68.4)
Ohmann	146/291 (50.2)
RIPASA	105/291 (36.1)
Modified Lindberg	102/291 (35.1)
Fenyö	101/291 (34.7)

**Table 3:** Accuracy statistics

CPRs	Negative likelihood ratio	Sensitivity*	Negative predictive value*
Pediatric CPRs			
LRAR (*n* = 262)	0.09 (0.04–0.25)	95.3 (87.9–98.5)	95.6 (88.5–98.6)
LRARR (*n* = 260)	0.07 (0.02–0.23)	96.5 (89.4–99.1)	96.5 (89.3–99.1)
PAS (*n* = 199)	0.07 (0.00–0.22)	95.3 (86.0–98.8)	96.7 (90.0–99.1)
Adult CPRs			
Christian (*n* = 242)	0.08 (0.00–0.22)	94.8 (86.5–98.3)	96.3 (90.2–98.8)
Lintula (*n* = 219)	0.50 (0.38–0.65)	54.0 (41.0–66.4)	83.2 (76.6–88.3)
MASS (*n* = 206)	0.30 (0.20–0.46)	73.0 (60.1–83.1)	88.2 (81.5–92.8)
Alvarado (*n* = 203)	0.03 (0.00–0.20)	98.4 (90.0–99.9)	98.8 (92.5–99.9)
MAS-Shera (n = 199)	0.03 (0.00–0.23)	98.4 (90.5–99.9)	98.5 (90.9–99.9)
Ohmann (*n* = 146)	0	100 (90.2–100.0)	100 (88.6–100.0)

Data is displayed as value (95% CI)

*Data is displayed as percentage (95% CI)

Table 4 presents numbers of patients with low suspicion of appendicitis according to each of the seven CPRs with a negative likelihood ratio < 0.1 in whom additional imaging was performed. In 30–46% of these patients, additional imaging studies had been performed during diagnostic work-up to exclude appendicitis. Nine patients had a false negative test result according to at least one of these CPRs and were diagnosed with appendicitis despite a low suspicion. In all nine patients, imaging showed inflammation of the appendix.

**Table 4:** Radiological examinations in patients with low suspicion of appendicitis according to the CPR

Low suspicion of appendicitis	Children who only underwent ultrasound	Children who underwent CT after ultrasound	Children who underwent MRI after ultrasound	Total number of children with low suspicion undergoing any radiological test	Children with appendicitis (false negative test result)
Ohmann (*N* = 38)	12 (32%)	0 (0%)	0 (0%)	12 (32%)	0 (0%)
Alvarado (*N* = 83)	27 (33%)	3 (4%)	0 (0%)	30 (36%)	1 (1%)
MAS-Shera (*N* = 67)	18 (27%)	1 (1%)	1 (1%)	20 (30%)	1 (1%)
PAS (*N* = 88)	30 (34%)	3 (3%)	2 (2%)	35 (40%)	3 (3%)
LRARR (*N* = 85)	32 (38%)	0 (0%)	0 (0%)	32 (38%)	3 (3%)
Christian (*N* = 108)	45 (42%)	3 (3%)	2 (2%)	50 (46%)	4 (4%)
LRAR (*N* = 91)	38 (42%)	2 (2%)	0 (0%)	40 (44%)	4 (4%)

Data is displayed as value (% of total patients with low suspicion of appendicitis per CPR)

## Discussion

The aim of this study was to investigate the value of CPRs in ruling out appendicitis in our retrospective cohort in terms of negative likelihood ratio in order to select CPRs that could potentially be included in a future prospective cohort study.

In this study, seven CPRs had a negative likelihood ratio point estimate < 0.1, which therefore could impact clinical decision-making.^19^ Therefore, these CPRs might be used in a future prospective cohort study comparing their ability to rule out appendicitis in children presenting with abdominal pain at the emergency department. Depending on the used CPR, in no more than 4% of the patients with a low suspicion of appendicitis, appendicitis was diagnosed within 30 days. In 30–46% of patients with a low suspicion of appendicitis, additional imaging studies had been undertaken.

Only a few studies have investigated the value of CPRs in ruling out appendicitis in children and they mostly expressed this value by sensitivity. The discriminatory power of a diagnostic test can best be displayed by likelihood ratios in our opinion, as it is not influenced by disease prevalence.^38^

Recent systematic reviews, comprising 10–12 prospective derivation and validation studies with a total of around 4000 children, investigated the Alvarado score and PAS in the pediatric population and found negative likelihood ratios for these CPRs that were similar to our results; for the Alvarado score, negative likelihood ratios between 0.03 (95% CI, 0–0.36) and 0.38 (95% CI, 0.21–0.70) were found. Regarding the PAS, negative likelihood ratios ranging between 0 and 0.27 (95% CI, 0.20–0.43) have been reported.^10,16^

Differences in negative likelihood ratios regarding the Alvarado score in the published negative likelihood ratios might be caused by different cutoff values that were used in the systematic reviews.^11,39^ Furthermore, daily practice concerning the use of additional imaging might differ between countries.^39^ Regarding the PAS, modest differences in negative likelihood ratio compared to the results in our study could be explained by the prospective nature of the included studies (versus our retrospective study) and by different inclusion criteria of the included population.

To our knowledge, we are the first to present negative likelihood ratios of other CPRs in addition to the Alvarado score and PAS in the same cohort. Furthermore, this study included multiple CPRs that do not incorporate extensive laboratory parameters. Multiple biochemical variables that are included in most CPRs, such as neutrophil count and leukocyte differentiation, are not routinely tested in the Netherlands when a child presents at the emergency department. Because of the identification and inclusion of both CPRs with and without extensive laboratory parameters, we were able to present a complete overview of all CPRs that can potentially be used in future prospective studies comparing their ability in ruling out appendicitis. In order to present a complete overview of all potential CPRs, we determined a low cutoff value of at least 50% of available data per CPR for inclusion in our analysis. We do realize that this cutoff value is low, but the aim of this study was to identify CPRs, investigate their potential in ruling out appendicitis, and investigate their appropriateness in the current diagnostic work-up as performed in the Netherlands in order to select them for a prospective cohort study. This cutoff value was determined prior to the identification of CPRs, and we realize that the use of a higher cutoff value might have led to a more stringent selection of only those CPRs that are most appropriate in our population.

The evidence-based guideline regarding the diagnosis and treatment of appendicitis, introduced by the Association of Surgeons of the Netherlands in 2010, emphasized reduction of the negative appendectomy rate. Imaging procedures are advocated to improve diagnostic accuracy and the consequence of this change has been the increased utilization of ultrasound as the initial imaging modality to evaluate abdominal pain in children in the Netherlands.^40^ In 2010, preoperative imaging procedures were performed in 44% of patients presenting at the emergency room with abdominal pain in the Netherlands, compared to only 22% of the patients a decade earlier.^3,41^ Currently in the Netherlands, in 99.7% of patients preoperative imaging is performed.^4^ A recent study conducted in the USA found that 99.7% of pediatric patients underwent preoperative-imaging studies as well.^42^ This differs significantly from the performance of preoperative imaging in the UK, where preoperative ultrasound and computed tomography (CT) were performed in 19.9 and 12.9% of patients respectively.^43^ Ultrasound has a high frequency of inconclusive results, reported to range between 37 to 51% in the pediatric population.^7,44^ Increased performance of ultrasound therefore results in increased use of costly and potentially harmful imaging studies, such as CT and MRI in pediatric patients.

In this study, in 30–46% of patients with a low suspicion of appendicitis according to these CPRs, additional imaging studies had been undertaken, whereas in no more than 4% of these patients (depending on the used CPR) acute appendicitis was diagnosed within 30 days. Nonetheless, because of the retrospective nature of this study, it might be possible that these additional imaging studies have not been solely performed to diagnose appendicitis, but also to exclude other potential diagnoses. Still, it raises the question whether or not watchful waiting should be considered for children with a low suspicion of appendicitis instead of additional imaging studies to rule out appendicitis. Opponents of this less aggressive diagnostic work-up mostly fear perforation of the appendix in case of complicated appendicitis.^45^ However, several studies have not found clinical observation or re-evaluation to be associated with a significantly higher incidence of complicated appendicitis and perforation.^46,47^ Time to presentation at the emergency department appears to be the main factor associated with perforation in children with appendicitis.^46,48^ Furthermore, literature suggests that perforation can rarely be prevented, implicating that a correct diagnosis is more important than a rapid treatment strategy.^49^

This study has several limitations. First, due to the single-center nature, generalizability might be reduced, although it was performed in a general teaching hospital. Second, the retrospective nature of this study might have led to selection bias and information bias. In case of wrong ICD code classification, patients might have been missed. We do realize that inclusion of 291 patients in 3 years’ time seems to be low for a large teaching hospital. This low number of patients could be explained by the fact that we only used ICD codes of acute appendicitis, acute abdomen, and general abdominal pain, because inclusion of children presenting with, for example, mainly symptoms of urinary tract infection would artificially decrease negative likelihood ratios and overestimate the value of the CPRs. Patient files with missing data were left out of the analysis. As a result, for only 34–90% of the patients the CPRs could be calculated. However, the aim of this study was not only to determine the ability of CPRs to rule out appendicitis in our cohort but also to investigate their appropriateness within the current diagnostic work-up as performed in the Netherlands. As mentioned previously, this study was performed in order to select appropriate and useful CPRs for a future prospective cohort study that can compare their value in ruling out appendicitis in our cohort. Nonetheless, missing data could have led to a selection bias, whereby the results of a CPR may have been inflated, leading to a low negative likelihood ratio. For example, in our population the CPR with the optimal negative likelihood ratio (Ohmann score) could only be calculated for 50.2% of the total population. Third, the small sample size causes wide confidence intervals for the calculated accuracy statistics.

In addition, CPRs are prone to subjective interpretation by the treating physician (e.g., variables of physical examination). In a prospective study by Mandeville, an interobserver agreement was found of 88 and 83.5% for the Alvarado score and the PAS, respectively.^48^ Another problem is the potential partial verification bias. Patients were classified in the non-appendicitis group if there was no recurrence of abdominal pain during 30 days after initial presentation. Patients who went to another hospital during these 30 days could have been missed. On the other hand, there is no nearby facility that is comparable to the general teaching hospital where this study was conducted. Therefore, it can be expected that patients attend the same emergency department as during their initial presentation. Another issue is that a number of patients were included in the appendicitis group despite the fact that intraoperative or histopathological findings were not obtained, potentially also leading to misclassification. However, these patients were included in the APAC study, in which a radiologically confirmed simple appendicitis was an inclusion criterion.

In conclusion, we identified seven CPRs that could potentially be used in a future prospective cohort study to compare their ability to rule out appendicitis in the pediatric population in the Netherlands and other countries with comparable diagnostic work-up. Further prospective studies are needed to investigate if imaging studies could safely be omitted and be replaced by clinical monitoring or re-evaluation in children with a low CPR score.

## Appendix 1

### Pubmed database search

((“Appendix”[Mesh] OR “Appendicitis”[Mesh] OR “Appendectomy”[Mesh] OR appendix[tiab] OR appendic*[tiab] OR appendec*[tiab]) AND (Clinical prediction rule*[tiab] OR prediction rule*[tiab] OR scoring system*[tiab])) AND (child*[tw] OR schoolchild*[tw] OR infan*[tw] OR adolescen*[tw] OR pediatri*[tw] OR paediatr*[tw] OR neonat*[tw] OR boy[tw] OR boys[tw] OR boyhood[tw] OR girl[tw] OR girls[tw] OR girlhood[tw] OR youth[tw] OR youths[tw] OR baby[tw] OR babies[tw] OR toddler*[tw] OR teen[tw] OR teens[tw] OR teenager*[tw] OR newborn*[tw] OR postneonat*[tw] OR postnat*[tw] OR perinat*[tw] OR puberty[tw] OR preschool*[tw] OR suckling*[tw] OR picu[tw] OR nicu[tw])

## Appendix 3 Clinical prediction rules

Alvarado score (cutoff value < 5: no appendicitis)

**Table:**

Variable	Points
Migration	1
Anorexia/acetone	1
Nausea/vomiting	1
Tenderness in right lower quadrant	2
Rebound pain	1
Elevation of temperature	1
Leukocytosis	2
Shift to the left	1
Total	10

Christian score (cutoff value < 3: no appendicitis)

**Table:**

Variable	Points
Abdominal pain	1
Vomiting	1
Right lower quadrant tenderness	1
Low grade fever (≤ 38.8 °C)	1
Polymorphonuclear leucocytosis (TC ≥ 10,000 with polymorphs ≥ 75%)	1
Total	5

Fenyö score (cutoff value ≤ − 17: no appendicitis)

**Table:**

Variable	Points
Constant	− 10
Male	8
Female	− 8
White blood cell count < 8.9	− 15
White blood cell count 8.9–13.9	2
White blood cell count > 14.0	10
Pain duration < 24 h	3
Pain duration 24–48 h	0
Pain duration > 48 h	− 12
Progression of pain	3
No progression of pain	− 4
Relocation of pain	7
No relocation of pain	− 9
Vomiting	7
No vomiting	− 5
Aggravation with cough	4
No aggravation with cough	− 11
Rebound tenderness	5
No rebound tenderness	− 10
Rigidity	15
No rigidity	− 4
Tenderness outside right lower quadrant	− 6
No tenderness outside right lower quadrant	4
Total	56

Lintula score (cutoff value ≤ 15: no appendicitis)

**Table:**

Variable	Points
Male	2
Female	0
Intensity of pain severe	2
Intensity of pain mild or moderate	0
Relocation of pain	4
Pain in the right-lower abdominal quadrant	4
Vomiting	2
Body temperature ≥ 37.5 °C	3
Guarding	4
Bowel sounds absent, tinkling, or high pitched	4
Rebound tenderness	7
Total	32

Low-Risk Appendicitis Rule

Low-Risk Appendicitis Rule Refinement.

Modified Alvarado Scoring System (MASS) (Cutoff value < 7: no appendicitis)

**Table:**

Variable	Points
Migratory right iliac fossa pain	1
Nausea/vomiting	1
Anorexia	1
Tenderness in right iliac fossa	2
Rebound tenderness in right iliac fossa	1
Elevated temperature	1
Leukocytosis	2
Total	9

Modified Lindberg score (cutoff value ≤ − 19: no appendicitis)

**Table:**

Variable	Points
Constant	− 10
Male	8
Female	− 8
White blood cell count < 12.0	− 15
White blood cell count 12.0–20.0	2
White blood cell count 20.0	10
Pain duration < 24 h	3
Pain duration 24–48 h	0
Pain duration > 48 h	− 12
Progression of pain	3
No progression of pain	− 4
Temperature ≥ 37.5 °C	7
Temperature < 37.5 °C	− 9
Vomiting	7
No vomiting	− 5
Migration of pain	4
No migration of pain	− 11
Rebound tenderness	5
No rebound tenderness	− 10
Rigidity	15
No rigidity	− 4
Tenderness outside right lower quadrant	− 6
No tenderness outside right lower quadrant	4
Total	56

Ohmann score (cutoff value < 6: no appendicitis)

**Table:**

Variable	Points
Tenderness, right lower quadrant	4.5
Rebound tenderness	2.5
No micturition difficulties	2.0
Steady pain	2.0
Leukocyte count ≥ 10.0 × 10^9^/L	1.5
Age < 50 years	1.5
Relocation of pain to right lower quadrant	1.0
Rigidity	1.0
Total	16.0

Pediatric Appendicitis Score (PAS) (cutoff value ≤ 5: no appendicitis)

**Table:**

Variable	Points
Cough/percussion/hopping tenderness	2
Anorexia	1
Pyrexia	1
Nausea/emesis	1
Tenderness in right lower quadrant	2
Leukocytosis	1
Polymorphonuclear neutrophilia	1
Migration of pain	1
Total	10

RIPASA score (cutoff value < 7.5: no appendicitis)

**Table:**

Variable	Points
Age < 40 years	1
Age > 40 years	0.5
Male	1
Female	0.5
Right iliac fossa pain	0.5
Migration of pain	0.5
Nausea and vomiting	1
Anorexia	1
Duration of symptoms < 48 h	1
Duration of symptoms > 48 h	0.5
Right iliac fossa tenderness	1
Guarding	2
Rebound tenderness	1
Rovsing’s sign	2
Fever	1
Raised white cell count	1
Negative urinalysis	1
Foreign national registration identity card	1
Total	16

## Appendix 4 Flow of participants for the CPRs

Alvarado score

Christian score

Lintula score

Low-Risk Appendicitis Rule

Low-Risk Appendicitis Rule Refinement

Modified Alvarado Scoring System

Modified Alvarado Score by Shera

Ohmann score

Pediatric Appendicitis Score
